# Supplementary material for: Multi‐dataset identification of innovative feature genes and molecular mechanisms in keratoconus
Source: J Cell Mol Med. 2024 Sep 19;28(18):e70079. doi: 10.1111/jcmm.70079 (PMC11412914; doi:10.1111/jcmm.70079)
Supplement: Supplementary file 2 — Material S2. [file JCMM-28-e70079-s002.zip › 7.GSEA analysis/3.DBNDD1/KEGG_BLADDER_CANCER.html]

Details for gene set KEGG\_BLADDER\_CANCER[GSEA]

|  || Dataset | DBNDD1.group.cls#h\_versus\_l |
| Phenotype | group.cls#h\_versus\_l |
| Upregulated in class | l |
| GeneSet | KEGG\_BLADDER\_CANCER |
| Enrichment Score (ES) | -0.41472507 |
| Normalized Enrichment Score (NES) | -1.1848409 |
| Nominal p-value | 0.3090909 |
| FDR q-value | 0.71050584 |
| FWER p-Value | 0.99 |
Table: GSEA Results Summary

  

Fig 1: Enrichment plot: KEGG\_BLADDER\_CANCER      
 Profile of the Running ES Score & Positions of GeneSet Members on the Rank Ordered List

  

| SYMBOL | TITLE | RANK IN GENE LIST | RANK METRIC SCORE | RUNNING ES | CORE ENRICHMENT || 1 | E2F2 | na | 6645 | 0.133 | -0.1550 | No |
| 2 | MMP2 | na | 7845 | 0.107 | -0.1603 | No |
| 3 | CCND1 | na | 9676 | 0.076 | -0.1922 | No |
| 4 | MDM2 | na | 9756 | 0.075 | -0.1739 | No |
| 5 | MAPK3 | na | 9865 | 0.073 | -0.1569 | No |
| 6 | RB1 | na | 10147 | 0.069 | -0.1459 | No |
| 7 | MAP2K2 | na | 10931 | 0.059 | -0.1522 | No |
| 8 | FGFR3 | na | 12198 | 0.045 | -0.1763 | No |
| 9 | ERBB2 | na | 13132 | 0.037 | -0.1929 | No |
| 10 | DAPK1 | na | 13939 | 0.030 | -0.2078 | No |
| 11 | TP53 | na | 13962 | 0.030 | -0.2001 | No |
| 12 | KRAS | na | 14127 | 0.029 | -0.1968 | No |
| 13 | TYMP | na | 14289 | 0.028 | -0.1938 | No |
| 14 | RPS6KA5 | na | 14808 | 0.024 | -0.2021 | No |
| 15 | BRAF | na | 15471 | 0.019 | -0.2158 | No |
| 16 | VEGFB | na | 17569 | 0.006 | -0.2747 | No |
| 17 | CDH1 | na | 18305 | 0.001 | -0.2957 | No |
| 18 | HRAS | na | 18984 | -0.004 | -0.3140 | No |
| 19 | RAF1 | na | 19005 | -0.005 | -0.3133 | No |
| 20 | EGF | na | 19333 | -0.007 | -0.3208 | No |
| 21 | DAPK3 | na | 20614 | -0.018 | -0.3526 | No |
| 22 | CDK4 | na | 21131 | -0.023 | -0.3611 | No |
| 23 | CDKN2A | na | 21295 | -0.025 | -0.3590 | No |
| 24 | EGFR | na | 22041 | -0.033 | -0.3715 | No |
| 25 | THBS1 | na | 22482 | -0.037 | -0.3740 | No |
| 26 | MYC | na | 22660 | -0.039 | -0.3684 | No |
| 27 | MAPK1 | na | 22705 | -0.040 | -0.3587 | No |
| 28 | RASSF1 | na | 23202 | -0.046 | -0.3604 | No |
| 29 | E2F3 | na | 24031 | -0.057 | -0.3687 | No |
| 30 | ARAF | na | 24817 | -0.068 | -0.3726 | No |
| 31 | DAPK2 | na | 26278 | -0.093 | -0.3890 | Yes |
| 32 | CXCL8 | na | 26911 | -0.106 | -0.3781 | Yes |
| 33 | NRAS | na | 27014 | -0.108 | -0.3514 | Yes |
| 34 | E2F1 | na | 27800 | -0.126 | -0.3393 | Yes |
| 35 | MAP2K1 | na | 29246 | -0.164 | -0.3357 | Yes |
| 36 | PGF | na | 29370 | -0.168 | -0.2931 | Yes |
| 37 | VEGFA | na | 30038 | -0.189 | -0.2602 | Yes |
| 38 | CDKN1A | na | 31724 | -0.250 | -0.2400 | Yes |
| 39 | VEGFC | na | 31739 | -0.251 | -0.1713 | Yes |
| 40 | MMP1 | na | 33729 | -0.395 | -0.1200 | Yes |
| 41 | MMP9 | na | 34473 | -0.541 | 0.0074 | Yes |
Table: GSEA details [plain text format]

  

Fig 2: KEGG\_BLADDER\_CANCER      
 Blue-Pink O' Gram in the Space of the Analyzed GeneSet

  

Fig 3: KEGG\_BLADDER\_CANCER: Random ES distribution      
 Gene set null distribution of ES for **KEGG\_BLADDER\_CANCER**
